# In-hospital mortality does not increase in patients aged over 85 years after hip fracture surgery. A retrospective observational study in a Japanese tertiary hospital

**DOI:** 10.1186/s40981-018-0172-3

**Published:** 2018-05-03

**Authors:** Yoshihisa Fujita, Kumi Shimada, Tomohiko Sato, Masahiko Akatsu, Koichi Nishikawa, Atsuko Kanno, Toshitake Aizawa

**Affiliations:** 1 Department of Anesthesia, Iwaki Kyoritsu General Hospital, 16 Kusehara, Mimaya-Machi, Uchigo, Iwaki, 973-8555 Japan; 2 Department of Disaster and Comprehensive Medicine, Fukushima Medical University, 1 Hikariga-oka, Fukushima, 960-1247 Japan; 3 Department of Orthopedic Surgery, Iwaki Kyoritsu General Hospital, 16 Kusehara Mimaya-Machi, Uchigo, Iwaki, 973-8555 Japan

**Keywords:** Hip fracture, Risk factors, Mortality, Surgery

## Abstract

**Introduction:**

Hip fracture is a common and serious orthopedic injury among the geriatric population, necessitating surgical treatment. We tested whether age is a significant risk factor for in-hospital mortality after surgery in this retrospective cohort study and, further, analyzed causes and pattern of death in those patients.

**Methods:**

We queried the electronic hospital records of in-patients aged over 75 years who had undergone hip fracture surgery from the start of 2010 to the end of August 2016 in our hospital, a tertiary hospital on the main island of Japan. The extracted data included patient ID, age, gender, location of fracture, ASA-PS scores, types of anesthesia, durations of anesthesia and surgery, days of hospital stay after surgery, and outcomes at hospital discharge including in-hospital death. The extracted data were divided into two groups based on the patient’s age at the time of surgery: the aged group (age of < 85) and the advanced age group (age of ≥ 85 years), and we compared patient characteristics and management variables and discharge disposition between the two groups.

**Results:**

Eight hundred four patient records were extracted (360 in the aged and 444 in the advanced age groups). Although a smaller proportion of patients in the advanced age group could be discharged home, all-cause in-hospital mortality was also similar between the two groups (1.9 and 1.6%, aged and advanced age groups, respectively). Six patients died from advanced cancer, and five patients died of pneumonia resulting from aspiration.

**Conclusions:**

The results of this study suggest that age is not a clinically significant risk factor for in-hospital mortality. The possibility decreasing in-hospital mortality exists in identifying patients at risk of aspiration and preventing it.

## Background

Hip fracture is a common and serious orthopedic injury among the geriatric population, necessitating surgical treatment. Reports from the UK and US indicate that 30-day postoperative mortality and morbidity are still high, at 6.5 and 5.2%, respectively [1, 2]. Although age is one of the significant predictors for increased mortality, it may actually be a surrogate measure for other variables, especially since frailty or reduced functional capacity rather than chronological age predict postoperative mortality after surgery overall [3–5]. Causes and pattern of death after hip fracture surgery are not well understood, and the need to improve postoperative mortality after hip fracture is clear.

In this study, we used the database of hip fracture patients in our hospital to analyze causes and pattern of death. We hypothesized that if age is a clinically significant factor for mortality after hip fracture surgery, there should be a difference between groups when stratified by chronological age.

## Methods

Iwaki Kyoritsu General Hospital Ethics Committee (the reference number H28-9) approved the study plan. We queried the electronic hospital records of in-patients aged over 75 years who had undergone hip fracture surgery from the start of 2010 to the end of August 2016 in our hospital, Iwaki Kyoritsu General Hospital. The ethics committee waived to obtain individual informed consent to participate in the study from patients.

This hospital is a tertiary hospital covering a population of 348,000 in an area of 1232 km^2^ on the main island of Japan. The extracted data included patient ID, age, gender, location of fracture, ASA score, types of anesthesia, durations of anesthesia and surgery, days of hospital stay after surgery, and outcomes at hospital discharge including in-hospital death. The primary outcome was all-cause in-hospital mortality.

The extracted data were divided into two groups based on the patient’s age at the time of surgery: the aged group (aged less than 85 years) and the advanced age group (aged 85 years or older), and we compared patient characteristics and management variables and discharge disposition between the two groups. We reviewed the electronic records of patients who died in the hospital following surgery to identify the cause of death, which was made from the clinical diagnosis. Currently, post-mortem examination is not usual in Japan when patients die after surgery during a hospital stay.

Statistical analysis was performed using SPSS Ver. 22. Univariate analyses between the aged and advanced age groups were performed using an unpaired *t* test for continuous variables, and chi-squared test for categorical data. We also used binomial multivariate logistic regression to test whether mortality (a dichotomous-dependent variable) can be predicted based on independent variables: ASA score, age groups, gender, types of anesthesia, and location of fracture. Descriptive data were presented as mean ± standard deviation (S.D.), and a *p* < 0.05 was considered significant.

## Results

### Comparison in-hospital mortality between the aged and advanced age groups

Demographic data showed some differences between two groups, and there was a slightly higher proportion of females and extra-capsular fractures in the advanced age group (Table 1). While ASA score did not differ between the two groups, a greater proportion of patients in the advanced age group underwent spinal anesthesia, and durations of anesthesia and surgery were shorter in the advanced age group than the aged group. There was no statistical difference in the length of hospital stay after surgery between the two groups. Neither mortality rate nor discharge disposition did not differ between two groups (Table 2). All-cause in-hospital mortality rates of the both groups were relatively low (1.9 and 1.6%, aged and advanced age groups respectively), and did not differ significantly. Binomial multivariate logistic regression analysis showed that only ASA score is an independent risk factor for in-hospital mortality. Other variables including age difference were not associated with mortality (Table 3).

**Table 1:** Comparative demographic characteristics of patients with hip fracture surgery between aged and advanced age groups

	Aged group (75 ≤ age < 85)	Advanced age group (85 ≤ age)	*p* value
	*n* = 360	*n* = 444	
Age (years)	80.4 ± 2.8	89.4 ± 3.5	0.000
Gender female	281 (78.1)	387 (87.2)	0.001
Locations of the fracture			0.008
Intra-capsular	157 (43.6)	161 (36.3)	
Extra-capsular	186 (51.7)	273 (61.5)	
others	17 (4.7)	10 (2.3)	
ASA-PS score			0.168
I or II	228 (58.6)	260 (63.3)	
III, IV, V	132 (41.4)	184 (36.7)	
Types of anesthesia			0.000
A	105 (29.2)	100 (22.5)	
B	134 (37.2)	98 (22.1)	
C	121 (33.6)	246 (55.4)	
Anesthesia duration (minutes)	110 ± 42	97 ± 33	0.001
Surgery duration (minutes)	61 ± 33	53 ± 24	0.001
LOS after surgery (days)	26.4 ± 18.7	22.4 ± 15.8	0.078

The data are given as number (%) or the mean ± S.D.

*ASA-PS* American Society of Anesthesiology physical status, *A* general anesthesia, *B* general anesthesia combined with neuraxial or regional block, *C* spinal anesthesia, *LOS* length of hospital stay

**Table 2:** Comparative discharge disposition with hip fracture surgery between aged and advanced age groups

	Aged group (75 ≤ age < 85)	Advanced age group (85 ≤ age)	*p* value
	*n* = 360	*n* = 444	
Outcomes			0.692
In-hospital death	7 (1.9)	7 (1.6)	
Disposition			0.174
Discharge to other hospitals/nursing home	201 (55.8)	277 (62.4)	
Discharge home	149 (41.4)	156 (35.1)	
others	3 (0.8)	4 (0.9)	

The data are given as number (%)

**Table 3:** In-hospital mortality after hip fracture surgery. Binominal multivariate regression analysis

Variables	β	SE	OR (95% CI)	p value
ASA-PS score	1.08	0.44	2.94 (1.23–7.03)	0.015
Age (85 ≤ age)	− 0.51	0.57	0.60 (0.20–1.81	0.599
Gender (female)	0.52	0.80	1.69 (0.35–8.14)	0.513
Location of fracture				0.717
Types of anesthesia				0.917

ASA-PS score was associated with in-hospital mortality after surgery. There was no association with other variables including age groups, gender, location of fracture and types of anesthesia

*Abbreviations: β* estimated regression coefficient, *SE* standard error of β, *OR* odds ratio, *95% CI* 95% confidence intervals for the odds ratio. *ASA-PS* American Society of Anesthesiology physical status explanation of variables, *ASA-PS score* continuous scale (I–V), *age* categorical (75 ≤ age < 85, 85 ≤ age), *types of anesthesia* categorical (general anesthesia, general anesthesia combined with neuraxial or regional block, spinal anesthesia), *location of fracture* categorical (inter-capsular, extra-capsular)

### Causes and pattern of death

Causes and pattern of death are summarized in the Table 4. There was no death related to anesthetic management. Six patients died from advanced cancer, all of which were diagnosed preoperatively, and five patients died of pneumonia resulting from aspiration. The other three patients died of pulmonary embolism, congestive heart failure, or sepsis. The mean length of hospital stay after surgery to death was longer in patients with advanced cancer than in patients with aspiration pneumonia (64.8 ± 45.7 and 19.4 ± 13.2 days, respectively). Because three patients in each group died later than 30 days after surgery, 30-day mortality rate was calculated as 0.9 and 1.1%, in the aged and advanced age groups, respectively, and it was assumed that patients discharged did not die within 30 days postoperatively.

**Table 4:** Causes and pattern of in-hospital mortality after hip fracture surgery

Causes	Number of patients	LOS (days)
Advanced cancer	6	64.8 ± 45.7
Aspiration pneumonia	5	19.4 ± 13.2
DVT/PE	1	12
CHF	1	25
Sepsis	1	10

*Abbreviations: LOS* length of hospital stay after surgery, *DVT* deep vein thrombosis, *PE* pulmonary embolism, *CHF* congestive heart failure

## Discussion

In this study, we found, firstly, that in-hospital mortality did not differ between aged and advanced age groups, which was confirmed by a univariate analysis and binominal multivariate logistic regression analysis, secondly, that in-hospital morality was low in both groups as compared to the reported values [1, 2], and, thirdly, that most patients died from advanced cancer or aspiration pneumonia.

There are several explanations for the absence of a difference in mortality between the two groups. First, the in-hospital mortality in this study was less than that previously reported from western countries. Even in those studies, the odds ratio of age among patients aged over 70 years is relatively small [1, 6]. Age should thus be considered as a risk only at the extremes of age, i.e., over 95 years [7]. Second, similarity of ASA-PS scores between the two groups may be an explanation, although we are not aware of the reason why there was no difference in ASA-PS scores between two groups. ASA-PS score, a well-recognized risk factor for postoperative mortality, which was also confirmed in the current study using the binominal multivariate regression analysis, generally increases in parallel with advancing age because the number and severity of co-morbidities also increase with aging. We therefore speculate that the ASA-PS score is the independent risk factor for the postoperative mortality in the patients with hip fracture and that age is a confounding variable having an effect on ASA-PS score. Lastly, longer hospital stays may have contributed to decrease mortality in both groups and hence resulted in the absence of mortality difference between two groups [8]. Many patients stayed in the hospital for more than 20 days after surgery in both groups. The longer hospital stays after surgery may reflect less efficient medical care in our health care system [9]. We also note that acute orthopedic and rehabilitation units are not clearly divided in most Japanese hospitals and postoperative patients often undergo rehabilitation while staying in orthopedic units, which may have better access for medical control.

Although anesthesia-related death is very rare, the best anesthetic techniques for hip fracture surgery are still a matter of debate [10]. In this study, spinal anesthesia was favored over general anesthesia in the advanced age group. We cannot attribute the low in-hospital mortality of the advanced age group to the choice of spinal anesthesia, however, because a mortality benefit with regional anesthesia (epidural or spinal anesthesia) is not proven [11]. We speculate that the attending anesthesiologists intended to avoid the postoperative delirium associated with general anesthesia in the advanced aged patients.

We found that most patients died from aspiration pneumonia or advanced cancer. The former may be regarded as preventable death. Many hip fracture patients show frailty, limited activities of daily living, and difficulty of swallowing on presentation. Visnjevac et al. [12] showed that the sub-classification based on their functional status is useful to predict postoperative mortality among octogenarian ASA-III patients. Preoperative assessment of these functions would be of help identifying patients at risk and thus prevent such complications after surgery. In their retrospective study, Chatterton et al. [1] also showed that the most common cause of death was respiratory infection. Preoperative identification of patients at highest risk for early death would help in tailoring surgical management and improving postoperative outcome, but further study is needed.

### Limitations

This is an observational study from a single center. Therefore, it is not necessarily representative of the whole Japanese hospital. However, we think that the results of this study reflect the average performance of tertiary hospitals in Japan because the quality of our hospital was acknowledged by the Japan Council for Quality Health Care. The observational design of our study also precludes causal conclusions. It is possible for patients discharged from the hospital to die earlier than 30 days after surgery at home or in nursing facilities, but such cases occur rarely, if at all. Because patients who are treated in tertiary hospitals are rarely discharged or transferred to another hospital, we believe that the 30-day mortality calculated from our cohort with longer hospital stays does not exceed the in-hospital mortality.

## Conclusions

This retrospective cohort study from a Japanese tertiary hospital demonstrated that in-hospital mortality after hip fracture surgery is relatively low and that it does not differ between the patients with age of < 85 and with age of ≥ 85 years, suggesting that age is not a clinically significant risk factor for in-hospital mortality. Most causes of in-hospital death were advanced cancer or aspiration pneumonia. The possibility decreasing in-hospital mortality exists in identifying patients at risk of aspiration and preventing it. Further studies are needed to assess and improve swallowing function in those patients.
